# Week-by-week changes in serum levels of bone-related circulating microRNAs and bone turnover markers

**DOI:** 10.1093/jbmrpl/ziae035

**Published:** 2024-03-22

**Authors:** Patryk Zarecki, Fatma Gossiel, Johannes Grillari, Miguel Debono, Matthias Hackl, Richard Eastell

**Affiliations:** School of Medicine and Population Health, Division of Clinical Medicine, University of Sheffield, S10 2RX Sheffield, United Kingdom; School of Medicine and Population Health, Division of Clinical Medicine, University of Sheffield, S10 2RX Sheffield, United Kingdom; Department of Biotechnology, Institute of Molecular Biotechnology, University of Natural Resources and Life Sciences, 1180 Vienna, Austria; Ludwig Boltzmann Institute for Traumatology, the Research Center in Cooperation with AUVA, 1200 Vienna, Austria; Austrian Cluster for Tissue Regeneration, 1200 Vienna, Austria; School of Medicine and Population Health, Division of Clinical Medicine, University of Sheffield, S10 2RX Sheffield, United Kingdom; Austrian Cluster for Tissue Regeneration, 1200 Vienna, Austria; TAmiRNA GmbH, 1110 Vienna, Austria; School of Medicine and Population Health, Division of Clinical Medicine, University of Sheffield, S10 2RX Sheffield, United Kingdom

**Keywords:** circulating microRNA, bone turnover markers, variability, osteoporosis, biomarkers, RT-qPCR

## Abstract

MicroRNAs are involved in post-transcriptional regulation of gene expression. Due to their regulatory role, microRNAs are differently expressed during specific conditions in healthy and diseased individuals, so microRNAs circulating in the blood could be used as diagnostic and prognostic biomarkers for various diseases and conditions. We want to investigate the variability of circulating microRNAs and bone turnover markers in weekly time intervals in older women. In a single-site longitudinal study, a panel of 19 bone-related miRNAs was measured using the osteomiR RT-qPCR assay in serum samples of 35 postmenopausal women divided into 3 groups: healthy controls (*n* = 12), low BMD (*n* = 14), and vertebral fractures (*n* = 9). Blood samples for measurement of CTX, PINP, OC, and bone ALP were collected once per week for 8 weeks at 9:00 AM after overnight fasting. Serum samples from all participants were analyzed for 19 microRNA bone biomarkers and 4 bone turnover markers over 8 weeks. We analyzed the data using a mixed model analysis of variance and found no significant changes between week-by-week time points in any of the groups. To estimate intraindividual variability between weekly time points, we have calculated the median coefficient of variation (CV). This was between 28.4% and 80.2% for microRNA, with an assay CV of 21.3%. It was between 8.5% and 15.6% for bone turnover markers, with an assay CV of 3.5% to 6.5%. The intraindividual variability was similar between groups. Circulating microRNAs measured in serum had a higher weekly intraindividual variability than bone turnover markers due in part to a higher assay CV.

## Graphical Abstract

**Graphical Abstract:**
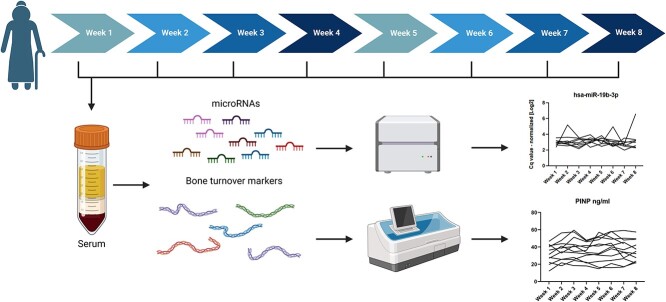

## Introduction

MicroRNAs (miRNAs) play a critical role in post-transcriptional regulation of gene expression. Due to the regulatory role, miRNAs are differently expressed during certain conditions in healthy and diseased individuals and could be used as diagnostic and prognostic biomarkers for various diseases and conditions. Previously published data have shown that some miRNAs can affect bone homeostasis, including bone remodeling and fracture healing, by altering the gene expression in osteoblasts, osteoclasts, and osteocytes.^1^ More than 2000 human miRNAs have so far been reported,^2^ which are transcribed in the nucleus as single-stranded RNAs, processed, and exported into the cytoplasm, where they are incorporated in the RNA-induced silencing complex to regulate gene expression. However, the active or passive release of miRNAs from cells within extracellular vesicles or bound to specific proteins results in a stable presence of miRNAs outside of cells, allowing the use of blood serum, urine, or saliva for detection.^3^ Such “circulating” miRNAs have been suggested as a promising source of minimal-invasive biomarkers^1^ in bone health and disease. Recent publications have considered the use of circulating miRNAs in fracture prediction and monitoring of osteoporosis treatment, as well as their use in secondary osteoporosis and rare bone disease.^1^

When making serial measurements of an analyte, it must show low day-to-day or week-to-week within-subject variability with the expected change. This issue of signal-to-noise has been studied extensively for bone turnover markers. For example, in monitoring alendronate therapy for osteoporosis, this ratio is highest for bone resorption markers, bone formation markers, and bone mineral density (BMD).^4^ In the EuBIVAS study, 91 subjects were studied weekly for 10 weeks, and the within-subject variability for CTX and PINP was 15% and 9%, respectively.^5^ The favorable ratio of signal to noise for bone turnover markers allows the identification of responders to bisphosphonate therapy in osteoporosis, as shown in the TRIO study,^6^ and this led the International Osteoporosis Foundation and International Federation of Clinical Chemistry and Laboratory Medicine (International Osteoporosis Foundation (IOF)/International Federation of Clinical Chemistry and Laboratory Medicine (IFCC)) to recommend the use of CTX and PINP for identifying poor adherence in patients started on oral bisphosphonate therapy for osteoporosis.^7^

We do not know whether the miRNA that are under study for osteoporosis have low variability and therefore likely to be useful in monitoring changes due to therapy. We need to know this for patients with osteoporosis (eg, low BMD or the presence of vertebral fracture) for our treatment studies and for women with normal BMD (for our prevention studies). There are limited data on the variability of miRNA in serum, and these tended to show large variability.^8^^,^^9^ There are no studies on osteoporosis. In the current study, we investigated week-to-week variability of miRNAs selected from previous studies as regulated in the context of bone^1^ and compared to bone turnover markers (BTMs) levels over 8 weeks in older women with and without osteoporosis. We hypothesize that miRNA varies more than BTM, and the variability is similar in women with and without osteoporosis.

## Material and methods

### Study design

We conducted a single site observational, longitudinal, case–control study of postmenopausal women within the SHATTER study (the Low Bone Mineral DenSity witH And wiThouT vErtebral fRactures).^10^^,^^11^ Levels of 19 endogenous circulating miRNAs specifically selected for associations with bone based on our previous research and literature, together with 3 synthetic miRNA controls were measured in the serum of postmenopausal women with low BMD and vertebral fractures, low BMD alone and healthy control group, all group matched for age and BMI.

### Subjects

We recruited 35 postmenopausal women from 3 groups: (1) healthy controls (*n* = 12); (2) patients with low BMD without any vertebral fractures (*n* = 14); (3) patients with low BMD and vertebral fractures before receiving treatment (*n* = 9).

BMD was measured at the LS in the posterior–anterior and lateral projections at a minimum of 2 unfractured lumbar vertebrae. Scans were acquired using the Hologic Discovery A densitometer (Hologic Inc.). Mean areal BMD (g/cm^2^) was calculated for vertebrae L1 to L4.

Vertebral fractures were identified on images from DXA-based vertebral fracture assessment (VFA) of the thoracolumbar spine using the Hologic Discovery A device. We used the algorithm-based qualitative assessment method to identify vertebral fractures and then graded their severity.^12^ Subjects with low BMD were defined by having a spine T-score < −1.0 (based on NHANES III.^13^ All groups were matched by age and BMI (Table 1).

**Table 1:** Clinical description of subjects included in the study.

	**Group 1** Healthy controls (*n* = 12)	**Group 2** Low BMD without vertebral fractures (*n* = 14)	**Group 3** Low BMD with vertebral fractures (*n* = 9)
**Age (yr)**	71.7 (7.8)	67.1 (5.8)	69.4 (6.6)
**Age at menopause (yr)**	47.4 (4.4)	49.4 (4.9)	52.2 (2.3)
**Weight (kg)**	77.2 (11.8)	67.9 (13.2)	68.1 (7.3)
**Height (m)**	1.61 (0.07)	1.60 (0.05)	1.61 (0.04)
**BMI (kg/m** ^**2**^**)**	29.8 (3.7)	26.5 (4.5)	26.3 (2.4)
**Spine BMD**	1.04 (0.09)	0,83 (0.05)	0,78 (0.09)
**Spine T-score**	0.13 (0.73)	−1,95 (0.49)	−2,50 (0.82)
**Fractures last year**	0%	0%	56%
**Non-vertebral fractures**	0%	0%	11%
**Relatives with osteoporosis**	17%	36%	44%
**Exercise**	92%	93%	78%
**Alcohol units per week**	3.8 (4.4)	1.8 (2.5)	6.2 (7.3)
**Smoker**	17%	36%	36%

### Sample collection

Blood samples were collected once per week for 8 weeks at 9:00 AM after overnight fasting and avoidance of intense exercise the previous day. Serum samples were stored at −80°C in the University of Sheffield Medical School Biorepository. Upon completion of the study, the samples were transferred to the South Yorkshire and North Derbyshire Musculoskeletal Biobank, which approved their use for measuring miRNA and BTMs. The South Yorkshire Research Ethics Committee approved the SHATTER study and all participants gave fully informed written consent before study enrolment.

### Bone turnover markers measurements

Serum C-terminal telopeptide of type 1 collagen (CTX) - CV 6.5%, procollagen type I N-propeptide (PINP) - CV 7.2%, osteocalcin (OC) - CV 6.3%, and bone alkaline phosphatase (BAP) - CV 3.5% were measured on the IDS-iSYS Multi-Discipline Automated Analyzer (Immunodiagnostic Systems).

### RNA extraction

The Serum/Plasma Kit (TAmiRNA GmbH) was used to perform RNA isolation. Serum samples frozen at −80°C were thawed on ice and centrifuged at 12000 *g* for 5 min at 4°C. After centrifugation, precisely 200 μL serum were mixed with 60 μL of lysis solution and 1 μL of RNA spike-in control by vortexing for 5 s. After incubation at room temperature for 3 min, 20 μL protein precipitation solution was added to the homogenized sample, vigorously vortexed, and incubated for 3 min at room temperature. After centrifugation at 12 000 *g* for 3 min at room temperature, exactly 200 μL of upper aqueous phase were taken and 2 μL glycogen were added to a final concentration of 5 mg/mL. RNA was precipitated with 200 μL of isopropanol. Samples were then transferred to columns, washed twice with wash solutions, once with 80% ethanol, and centrifuged for 5 min at 12000 *g* to clear out the columns from wash solution and ethanol residues. RNA was eluted with 30 μL of nuclease-free water, and stored at −80°C.

### qPCR analysis

About 2 μL of isolated RNA were used for reverse transcription to obtain cDNA, using the osteomiR chemistry Kit (TAmiRNA GmbH). Synthetic cDNA spike-in (cel-miR-39) was added as a spike-in control to monitor for enzyme inhibition during reverse transcription reaction. The reaction was incubated at 42°C for 60 min, and then heat inactivated at 95°C for 5 min. cDNA samples were stored at −20°C. Reverse transcription quantitative PCR (RT-qPCR) analysis of 21 circulating miRNAs and 3 miRNA spike-in controls was conducted using osteomiR 384-well panels (TAmiRNA GmbH). For RT-qPCR analysis, cDNA samples were diluted 50-fold and 5 μL were used in individual 10 μL PCR reactions using miGreen SYBR Green master mix and LNA-enhanced miRNA primer assays together with a PCR spike-in control (TAmiRNA GmbH). RT-qPCR program was set for: 95°C for 10 min initial denaturation, 45 cycles of denaturation (95°C, 10 s) and annealing (60°C, 60 s), and melting curve analysis on LightCycler 480 Real-Time PCR machine (Roche). To calculate the cycle of Cq-values, the second derivative method was used.

### miRNA expression analysis

The set of 21 circulating miRNAs, including 2 hemolysis controls, was selected based on the literature and our previous studies.^1^ We studied the following miRNAs: hsa-miR-375, hsa-miR-532-3p, hsa-miR-19b-3p, hsa-miR-152-3p, hsa-miR-23a-3p, hsa-miR-335-5p, hsa-miR-21-5p, hsa-miR-486-3p, hsa-miR-30e-5p, hsa-miR-127-3p, hsa-miR-214-3p, hsa-miR-550a-3p, hsa-miR-106b-5p, hsa-miR-133b, hsa-miR-143-3p, hsa-miR-144-3p, hsa-miR-451a, hsa-miR-29b-3p, hsa-miR-96-5p, and hsa-miR-188-5p. These miRNAs were chosen as they relate to bone metabolism; for example, has-miR-19b-3p modulates the estrogen signaling pathway, has-miR-29b-3p regulates the synthesis of extracellular matrix, has-miR-152-3p modulates ECM-receptor interaction, has-miR-152-3p modulates the signaling pathway regulating pluripotency of stem cells, and has-miR-21-5p regulates Hippo signaling pathway.^11^ Also, we have found some of them associated with osteoporosis in our previous studies.^11^^,^^14^

The quality of data for each sample was tracked using synthetic miRNA spike-in controls during each step of the workflow: RNA-Isolation (UniSp4); cDNA synthesis (cel-miR-39-3p); and RT-qPCR amplification (UniSp3). Having a quantitative quality control at each step allowed us to ensure that only samples with homogeneous purification and enzyme efficiency were considered for the final analysis. Technical variation was reduced by normalizing data against UniSp4 using the following equation:

normalized Cq = Cq UniSp4 - Cq measured miRNA

The presence of hemolysis in serum samples was controlled by calculating the ratio of miR-23a-3p and miR-451a, calculated with the equation:

hemolysis ratio = Cq hsa-miR-23a-3p - Cq hsa-miR-451a

miR-23a-3p is known to be low abundant in red blood cells, while miR-451a is highly enriched and therefore changes according to the degree of hemolysis.^15^

Samples with hemolysis ratio higher than 7 have been excluded from further analysis.

### Statistical analysis

Statistical analysis for groups comparison was performed using GraphPad Prism (GraphPad Software) by a mixed model analysis of variance approach with statistical significance of *P*-value: *P* < .05 as ^*^; *P* < .01 as ^**^; *P* < .001 as ^***^; *P* < .0001 as ^****^.

To estimate intraindividual variability between weekly time-points, we have calculated the median coefficient of variation (CV). We chose to report medians as the distribution of CVs is not normally distributed.

## Results

### Subject exclusion

The set of 21 specifically selected endogenous miRNAs, including 2 miRNAs for the monitoring of hemolysis and 3 synthetic spike-in controls were measured in 8 serum samples of 35 postmenopausal women (total 280 samples).

No sample was excluded because of hemolysis. After spike-in quality control, none of the samples was removed from further analysis due to inhomogeneous reverse transcription or PCR amplification.

### Clinical characteristics

A total of 35 postmenopausal women (age, mean [SD], 69.3 [6.8] yr, weight = 71.1 [12.0] kg and height = 160.0 [5.0] cm) were included in the analysis (Table 1). Of these, 12, 14, and 9 women were recruited to Groups 1, 2, and 3, respectively. In Group 3, 11 vertebral fractures were identified using the algorithm-based qualitative approach. Eight women had sustained one vertebral fracture and one woman had sustained 3 vertebral fractures (Table 2). All the study’s vertebral fractures were most prevalent at the thoracolumbar junction (T12 to L1). We observed wedge (64%) and concave/biconcave (36%), and no compression fractures. Of all the identified fractures, 9% were categorized as grade 1, 55% as grade 2, and 36% as grade 3. None of the patients from group 3 was being treated for osteoporosis.

**Table 2:** Vertebral fractures characteristics.

**ID**	**Number of fractures**	**Location, grade, description**
**VF1001**	1 of 1	T7, grade 1, concave
**VF1007**	1 of 1	T12, grade 2, wedge
**VF1018**	1 of 1	L1, grade 3, concave
**VF1025**	1 of 1	T11, grade 2, wedge
**VF1029**	1 of 1	T7, grade 2, biconcave
**VF1031**	1 of 1	T7, grade 3, wedge
**VF1033**	1 of 3	T7, grade 2 wedge
	2 of 3	T10, grade 2, concave
	3 of 3	T11, grade 3, wedge
**VF1034**	1 of 1	T10, grade 2, wedge
**VF1035**	1 of 1	T7, grade 3 wedge

**Table 3:** Inter-individual changes in miRNAs and BTMs levels (median CV%).

**miRNA**	**Healthy controls**	**Low BMD**	**Vertebral fractures**
**hsa-miR-375**	41.7%	53.8%	47.0%
**hsa-miR-214-3p**	80.2%	62.7%	61.4%
**hsa-miR-550a-3p**	29.1%	46.9%	34.6%
**hsa-miR-486-3p**	31.8%	52.1%	34.3%
**hsa-miR-144-3p**	39.7%	49.1%	43.1%
**hsa-miR-532-3p**	36.4%	40.4%	29.8%
**hsa-miR-30e-5p**	35.4%	42.3%	34.1%
**hsa-miR-19b-3p**	28.4%	41.0%	29.5%
**hsa-miR-106b-5p**	33.4%	47.2%	35.5%
**hsa-miR-133b**	52.7%	57.6%	49.5%
**hsa-miR-152-3p**	46.9%	50.2%	42.6%
**hsa-miR-127-3p**	71.9%	77.9%	49.0%
**hsa-miR-451a**	34.4%	41.0%	50.3%
**hsa-miR-23a-3p**	55.6%	49.3%	48.4%
**hsa-miR-29b-3p**	36.6%	42.5%	43.3%
**hsa-miR-143-3p**	50.1%	54.2%	39.1%
**hsa-miR-335-5p**	53.8%	57.6%	55.5%
**hsa-miR-21-5p**	41.0%	44.5%	38.0%
**hsa-miR-96-5p**	45.8%	46.8%	49.9%
**hsa-miR-188-5p**	43.0%	33.8%	38.5%
**BTM**	**Healthy controls**	**Low BMD**	**Vertebral fractures**
**CTX**	13.4%	16.8%	17.9%
**PINP**	13.6%	13.2%	15.6%
**OC**	12.7%	8.5%	11.8%
**BAP**	9.2%	9.8%	11.1%

### miRNA expression analysis

We have measured serum levels of 21 miRNAs in the samples of 35 postmenopausal women at weekly intervals for 8 weeks.

We analyzed the data using a mixed model analysis of variance approach and we found no significant changes between week-by-week time-points in any of groups.

**Figure 1:**
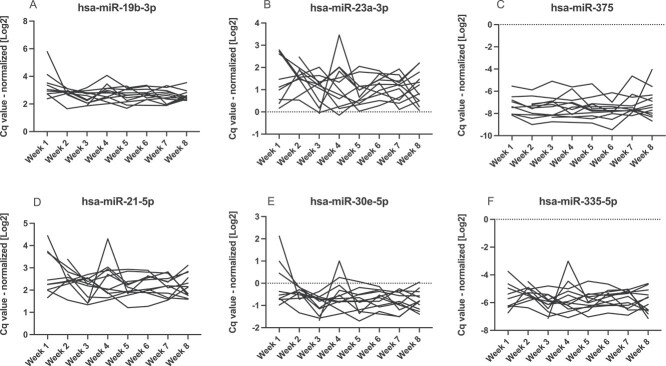
Intraindividual changes in miRNA serum levels (A-F) – healthy controls (*n*=12). Serum levels of selected miRNAs were measured in the healthy control group using RT-qPCR. Cq-values were normalized to UniSP4 spike-in control. The peak seen at week 4 was from the same subject, but no cause for the peak was identified

**Figure 2:**
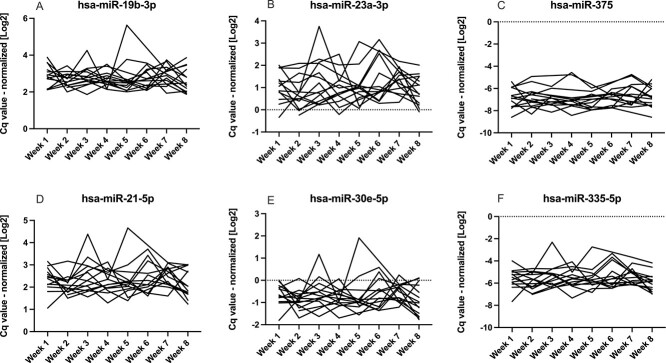
Intraindividual changes in miRNA serum levels (A-F) – low BMD (*n*=14). Serum levels of selected miRNAs were measured in the low BMD group using RT-qPCR. Cq-values were normalized to UniSP4 spike-in control.

**Figure 3:**
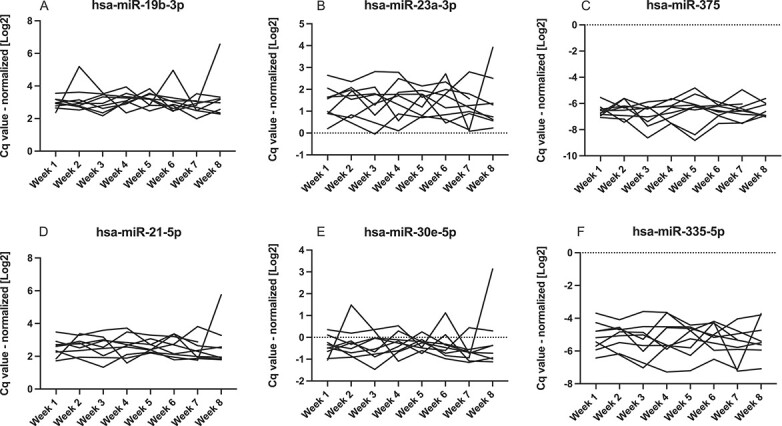
Intraindividual changes in miRNA serum levels (A-F) – vertebral fractures (*n*=9). Serum levels of selected miRNAs were measured in the vertebral fractures group using RT-qPCR. Cq-values were normalized to UniSP4 spike-in control.

In the healthy control group, miRNAs median intraindividual (within-subject) CV were between 28.4% (hsa-miR-19b-3p) and 80.2% (hsa-miR-214-3p); in low BMD group, 33.8% (hsa-miR-188-5p) and 77.9% (hsa-miR-127-3p); in vertebral fractures group, 29.5% (hsa-miR-19b-3p) and 61.4% (hsa-miR-214-3p) (Table 3, Figures 1–3). Combined assay CV, including RNA isolation, reverse transcription, and RT-qPCR, was 21.3% (Table 4).

**Figure 4:**
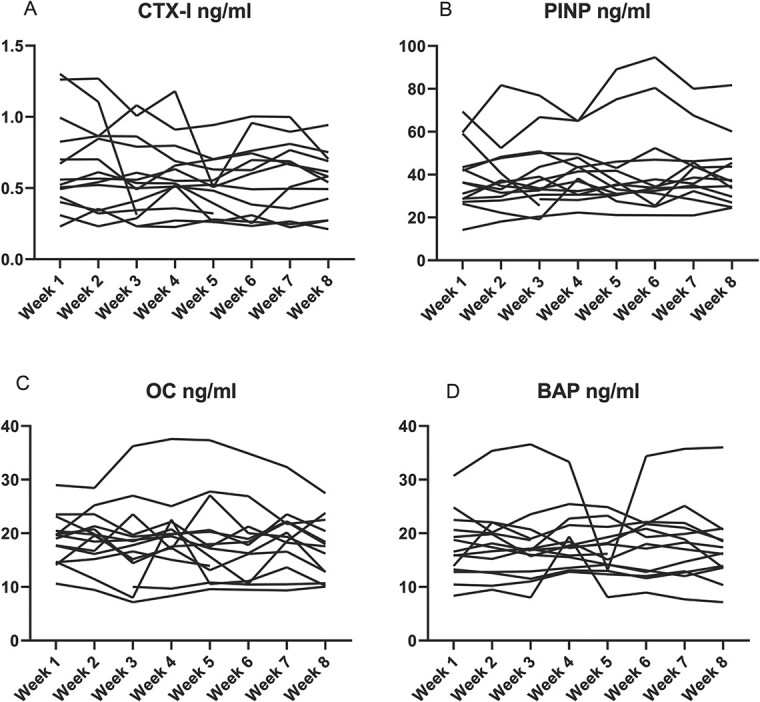
Intraindividual changes in serum levels of bone turnover markers (A - CTX-I, B - PINP, C - OC, D - BAP) – healthy controls (*n*=12). Bone turnover markers was measured in the healthy control group on the IDS-iSYS Multi-Discipline Automated Analyzer. Blood samples were collected once per week for 8 weeks at 9:00 AM after overnight fasting and avoidance of intense exercise the previous day.

**Figure 5:**
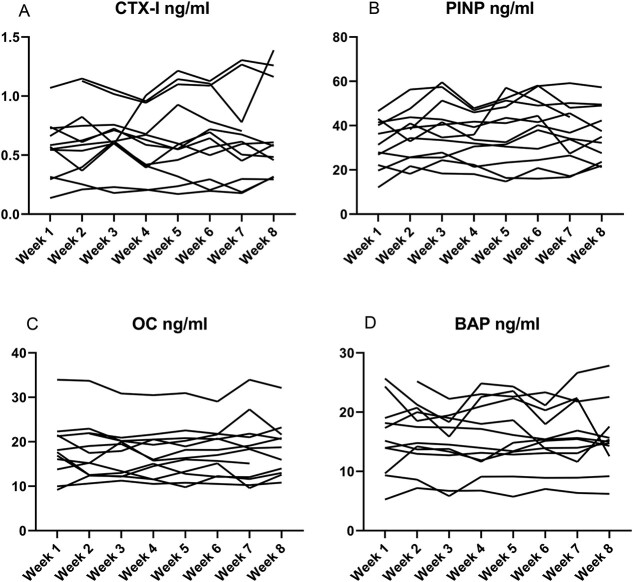
Intraindividual changes in serum levels of bone turnover markers (A - CTX-I, B - PINP, C - OC, D - BAP) – low BMD (*n*=14). Bone turnover markers was measured in the low BMD group on the IDS-iSYS Multi-Discipline Automated Analyzer. Blood samples were collected once per week for 8 weeks at 9:00 AM after overnight fasting and avoidance of intense exercise the previous day.

**Figure 6:**
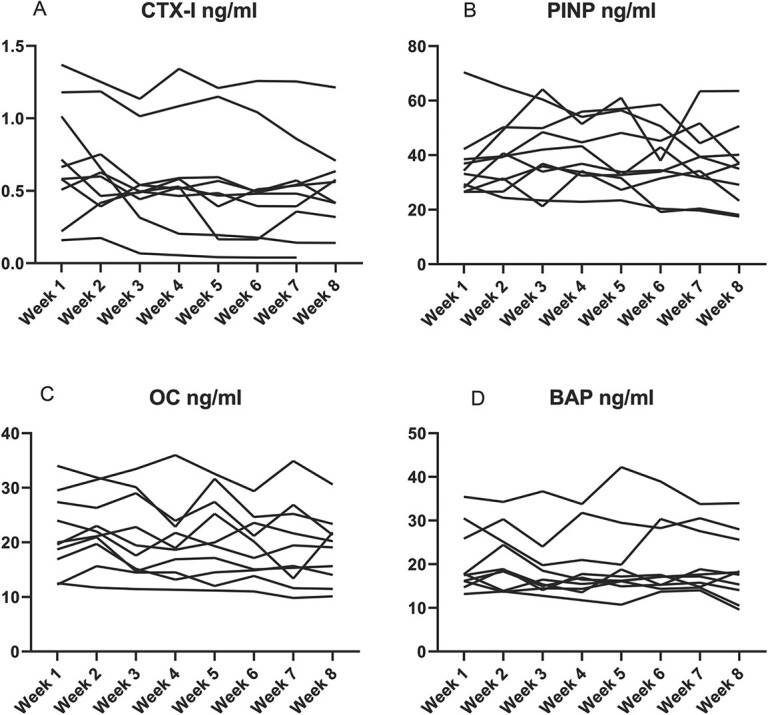
Intraindividual changes in serum levels of bone turnover markers (A - CTX-I, B - PINP, C - OC, D - BAP) – vertebral fractures (*n*=9). Bone turnover markers were measured in the vertebral fractures group on the IDS-iSYS Multi-Discipline Automated Analyzer. Blood samples were collected once per week for 8 weeks at 9:00 AM after overnight fasting and avoidance of intense exercise the previous day.

CTX intraindividual median CV was 13.4% in the healthy control group, 16.8% in the low BMD group, and 17.9% in the vertebral fractures group (Table 3, Figures 4–6), with assay CV of 6.5% (Table 4).

PINP intraindividual median CV was 13.6% in the healthy control group, 13.2% in the low BMD group, and 15.6% in the vertebral fractures group (Table 3, Figures 4–6), with assay CV of 7.2% (Table 4).

Osteocalcin intraindividual median CV was 12.7% in the healthy control group, 8.5% in the low BMD group, and 11.8% in the vertebral fractures group (Table 3, Figures 4–6), with assay CV of 6.3% (Table 4).

Bone alkaline phosphatase intraindividual median CV was 9.2% in the healthy control group, 9.8% in the low BMD group, and 11.1% in the vertebral fractures group (Table 3, Figures 4–6), with assay CV of 3.5% (Table 4).

We calculated the intraindividual variability for CTX (and the key bone turnover marker) and miRNA-21-5p (the most promising miRNA based on our previous paper).^11^ We tested the association by Spearman rank correlation, and the coefficient was not significant at 0.15. Thus, those subjects with the most variability in miRNA were not the same subjects most variable with CTX.

## Discussion

The week-to-week within-subject variability for miRNA was higher in all groups than for BTM. One cause of the higher variability was the assay CV. This was 21% for miRNA, much higher than the 3%–7% for BTM. Previous studies of variability for miRNAs gave assay variability of 6% to 69%.^16^

These estimates of long-term CV for miRNA are similar to those published in a study that showed a proportion of miRNAs are particularly “noisy,” more so in serum than plasma.^17^ There is no study of the variability of miRNAs in serum over several weeks analyzed in the same way as in the present study, but some work with longer intervals, for example, annually,^18^ (19) or in cerebrospinal fluid,^19^ and these showed that some, but not all miRNAs show a significant degree of variability.

The next step is to reduce the assay CV for miRNAs, which may be caused by (i) manual assay handling (compared to automated BTM analysis) and (ii) multiple processing steps (RNA isolation, reverse transcription, PCR) resulting in an accumulation of variance. This could be achieved by switching to detection technologies such as antibody-based detection,^20^ which show limited sensitivity.

**Table 4:** Inter-assay coefficient of variation.

**miRNA assay**		**SD**	**CV%**
**RT-qPCR (**UniSp3)		0.124	9.0%
**Reverse transcription (**cel-miR-39-3p)		0.212	15.9%
**RNA isolation (**UniSp4)		0.279	21.3%
**BTM assay**	**CV%**		
**CTX**	6.5%		
**PINP**	7.2%		
**OC**	6.3%		
**Bone ALP**	3.5%		

To estimate assay variability, we measured the levels of synthetic spike-in controls over the course of 8 weeks in each sample. We calculated a median coefficient of variation (CV%) using spike-in controls added during each step of the workflow. UniSp4 was added at the beginning of the RNA isolation protocol and was present in samples throughout the whole workflow. Cel-miR-39-3p was added to monitor for enzyme inhibition during reverse transcription reaction. UniSp3 was added before the RT-qPCR reaction to compare reaction efficiency between each sample.

The assay CVs for BTM in the present study are similar to what has been reported. In the Vasikaran^21^ position paper, the estimates for CTX and PINP were up to 6%. In the EuBIVAS study, the CVs for CTX and PINP were 5.0% and 3.6%, a little lower than the values of 6.5% and 7.2% in the present study; however, EuBIVAS excluded 2%–4% of samples as they were outliers or affected the variance homogeneity analyses.^5^

The within-subject CV for BTM was similar to those published. Vasikaran^21^ in an IOF/IFCC Position Paper summarized the data for CTX and PINP as up to 10% and 9%, and the EuBIVAS study estimated for CTX and PINP as 15.1% and 8.8%,^5^ so the values in the present study for CTX of 13 to 18, and for PINP of 13% to 16% are similar, if not identical.

The sources of variability of bone turnover markers have been studied thoroughly.^22^ Thus, for CTX, there can be variability within an individual due to the type of sample (serum or plasma), due to a recent meal (lower values), sampling in the afternoon rather than the morning (lower values), intense exercise (higher values), and the presence of diseases or the use of drugs. To reduce these sources of variability, it is usually recommended that CTX is taken from a fasting subject in the morning and that intense exercise is avoided the previous day, as was done in this study. The same determinants of variability for BTM are likely present for miRNAs: sample processing is a major source of variability evidenced by the large differences in miRNA profiles between serum and plasma,^23^ and the impact of plasma processing conditions.^24^ Furthermore, circadian rhythm,^24^ feeding effect, and day-to-day variability need further study.

The effects of osteoporosis treatments on BTMs are well described. The most standard treatments for osteoporosis are bisphosphonates. The percent decrease in BTMs is much greater than the within-subject variability in most subjects with postmenopausal osteoporosis and the ratio is referred to as the signal-to-noise ratio.^4^ The within-subject variability can be used to calculate the least significant change to identify responders.^25^ More than 80% of patients can be considered responders after 12 weeks of treatment with oral bisphosphonates.^6^

There is only preliminary information about osteoporosis therapy’s effects on miRNAs, due to which the magnitude of the response has yet to be discovered, for example, the effect of bisphosphonate therapy on miRNA in man. There is some evidence for changes in miR-33-3p and mir-133a in response to teriparatide, as well as increases in miR-26b-5p and miR-454-3p of 100% to 200%,^26^ although no change was reported by others.^27^ The need for more consistency in these studies is likely explained by the high variability in miRNA measurements and points to the need to have studies that are sufficiently powered. These clinical studies were conducted in women with low BMD with or without vertebral fracture, so the information included in the present paper is relevant to these. Once we have this information, we can evaluate the signal-to-noise ratio for miRNA monitoring of osteoporosis treatment.

In conclusion, circulating miRNAs measured in serum have a higher weekly intraindividual variability than bone turnover markers due partly to a higher assay CV. Strategies are required to reduce this variability. The high variability needs to be considered when designing future studies that include miRNAs.

## Data Availability

Data are available from the authors on reasonable request as part of a collaboration project.
